# Hepatocyte Differentiation from iPSCs or MSCs in Decellularized Liver Scaffold: Cell–ECM Adhesion, Spatial Distribution, and Hepatocyte Maturation Profile

**DOI:** 10.1080/15476278.2022.2061263

**Published:** 2022-04-17

**Authors:** Radiana Dhewayani Antarianto, Adrian Pragiwaksana, Wahyunia Likhayati Septiana, Nuzli Fahdia Mazfufah, Ameer Mahmood

**Affiliations:** a Department of Histology, Faculty of Medicine, Universitas Indonesia, Jakarta, Indonesia; b Stem cell and tissue engineering research cluster, (IMERI) Indonesian Medical Education and Research Institute, Jakarta Indonesia; c Program Doktor Ilmu Biomedik, Faculty of Medicine, Universitas Indonesia, Jakarta, Indonesia; d Program Master Ilmu Biomedik, Faculty of Medicine, Universitas Indonesia, Jakarta, Indonesia; e Department of Histology, Faculty of Medicine, Universitas Gunadarma, Depok, Indonesia; f Stem cell unit Department of Anatomy, King Saud University, Riyadh, Kingdom Saudi Arabia

**Keywords:** Hepatocyte, differentiation, iPSCs, MSCs, decellularized-liver-scaffold

## Abstract

Mesenchymal stem cells (MSC) and induced pluripotent stem cells (iPSC) have been reported to be able to differentiate to hepatocyte in vitro with varying degree of hepatocyte maturation. A simple method to decellularize liver scaffold has been established by the Department of Histology, Faculty of Medicine, Universitas Indonesia, in SCTE IMERI lab.^15^ This study aims to evaluate hepatocyte differentiation from iPSCs compared to MSCs derived in our decellularized liver scaffold. The research stages started with iPSC culture, decellularization, seeding cell culture into the scaffold, and differentiation into hepatocytes for 21 days. Hepatocyte differentiation from iPSCs and MSCs in the scaffolds was characterized using hematoxylin–eosin, Masson Trichrome, and immunohistochemistry staining to determine the fraction of the differentiation area. RNA samples were isolated on days 7 and 21. Expression of albumin, CYP450, and CK-19 genes were analyzed using the qRT-PCR method. Electron microscopy images were obtained by SEM. Immunofluorescence examination was done using HNF4-α and CEBPA markers. The results of this study in hepatocyte-differentiated iPSCs compared with hepatocyte-differentiated MSCs in decellularized liver scaffold showed lower adhesion capacity, single-cell-formation and adhered less abundant, decreased trends of albumin, and lower CYP450 expression. Several factors contribute to this result: lower initial seeding number, which causes only a few iPSCs to attach to certain parts of decellularized liver scaffold, and manual syringe injection for recellularization, which abruptly and unevenly creates pattern of single-cell-formation by hepatocyte-differentiated iPSC in the scaffold. Hepatocyte-differentiated MSCs have the advantage of higher adhesion capacity to collagen fiber decellularized liver scaffold. This leads to positive result: increase trends of albumin and higher CYP450 expression. Hepatocyte maturation is shown by diminishing CK-19, which is more prominent in hepatocyte-differentiated iPSCs in decellularized liver scaffold. Confirmation of mature hepatocyte-differentiated iPSCs in decellularized liver scaffold maturation is positive for HNF4-a and CEBPA. The conclusion of this study is hepatocyte-differentiated iPSCs in decellularized liver scaffold is mature with lower cell–ECM adhesion, spatial cell distribution, albumin, and CYP450 expression than hepatocyte-differentiated MSCs in decellularized liver scaffold.

## Introduction

Liver transplantation is the mainstream medical therapy for patients with end-stage liver disease.^1^ Significant restriction in liver transplantation is the limited number of available and suitable living liver donors. Liver transplant failure could be due to acute or chronic rejection. Standard post-transplant regiment to overcome rejection is the long-term use of immunosuppressive drugs. This increases the risk of severe viral or fungal infection in addition to malignancy. In brief, hepatocyte replacement therapy uses isolated hepatocytes of liver resected from a relative. They are expanded in vitro before being infused to the patient. This procedure has been shown to reduce immunosuppressive drug use and related complication.^2^

The problem with hepatocyte replacement therapy is the low hepatocytes yield due to cell proliferation arrest in culture. Stem cells have higher capacity to meet the expected number. Mesenchymal stem cells (MSC) and induced pluripotent stem cells (iPSC) are able to differentiate to hepatocyte in vitro with varying degree of hepatocyte maturation.^3–10^

In a study conducted by Blackford et al.,^11^ validation of current Good Manufacturing Practice (cGMP) in vitro hepatocytes differentiation from iPSCs using 2D monolayer culture or 3D culture with with differentiation induction medium. The medium comprises basal medium, cytokine, growth factors, and small molecules. It is switched periodically to recapitulate stages of in vivo hepatocyte development. The first differentiation stage starts from pluripotent or multipotent stem cells, which become definitive endodermal cells by using Activin A/transforming growth factor β (TGF-β). At this stage, increase in Wnt/β-catenin signaling pathway is important. The second stage occurs when the definitive endoderm differentiates into hepatoblast by using bone morphogenetic protein 4 (BMP4) and fibroblast growth factor (FGF) to induce the expression of specific liver genes. The third stage is differentiation from hepatoblasts to hepatocytes using hepatocyte growth factor (HGF), oncostatin M (OSM), dexamethasone, and/or 3D reaggregation with other cell types to specify their fate as hepatocytes.^12,13^

A simple method to decellularize liver scaffold has been established and characterized by the Department of Histology, Faculty of Medicine, Universitas Indonesia, in SCTE IMERI lab.^14,15^ This study aims to compare the profile of hepatocyte differentiation from iPSCs or MSCs in decellularized liver scaffold. The research was conducted from January 2020 to June 2021 at the Stem Cell and Tissue Engineering (SCTE) laboratory of IMERI FKUI, Histology laboratory, Center laboratory for Materials and Processing Failure Analysis (CMPFA) Metallurgical Engineering FTUI, Molecular Laboratory Biology and Proteomic Core Facilities (MBPCF) IMERI FKUI, and the Human Genetic Research Center (HGRC) laboratory of IMERI FKUI.

## Materials and methods

### Materials

MSC cell line from human umbilical cord Wharton jelly was developed in SCTE IMERI FKUI laboratory. Complete MSC medium included 10% PRP, 1% heparin (1000 U/ml), 1% amphotericin B (250ug/ml), 1% pen-strep (10.000 U/ml penicillin and 1000 ug/ml streptomycin), and αMEM.

iPSC cell line from human bone marrow mesenchymal stem cells was purchased from EBiSC, UK, catalog number UKKi006-A. Complete iPSC medium included Essential 8 medium and supplement 50X and plate coated with Vitronectin Recombinant Human Protein Gibco^TM.S^. Harvest reagents included Dulbecco’s phosphate-buffered saline (DPBS), ethylenediaminetetraacetic acid (EDTA), and rho-associated protein kinase inhibitor (ROCK inhibitor).

Liver scaffold decellularization included sodium dodecyl sulfate (SDS) 1^st^ BASE, Triton X-100 10%, EGTA, aquabides, NaCl 0.9%, and NaOH.

Hepatocyte differentiation induction medium included RPMI-1640, human serum albumin (HSA) 10%, Abam 1%, Glutamax 1%, Hepatozyme, and fetal bovine serum (FBS) 10%. Small molecules and growth factor for hepatocyte differentiation included 1.5 μM CHIR9901, 5 ng/ml bone morphogenetic protein 4 (BMP4), 5 μM LY29004, 40 ng/ml fibroblast growth factor 2 (FGF2), 50 ng/ml Activin A dan 25 ng/ml, 5 ng/ml Oncostatin *M* (OSM), dan 25 ng/ml hepatocyte growth factor (HGF).

HE staining: formaline 10%, alcohol 70%, alcohol 80%, alcohol 96%, alcohol 100%, xylol, paraffin wax, Hematoxylin dye, Eosin dye, aquadest, entellan. Masson Trichrome staining: Bouin’s solution, Weigert Hematoxylin, biebrich scarlet, phosphomolybdic acid, aniline blue. Immunohistochemistry: hydrogen peroxidase 30%, methanol, Recombinant Anti-Albumin antibody [EPSISR1] (ab240109 Abcam, UK), Recombinant Anti-Cytochrome P450 *3A4/CYP3A4 antibody* [EPR6202] (ab245774 Abcam, UK), Rabbit specific HRP/DAB Detection IHC Detection Kit Micro-polymer (ab236469 Abcam, UK), paraffin embedded rat liver slide from the Department of Histology, Faculty of Medicine, Universitas Indonesia lab, frozen rabbit liver stored in −20° freezer from SCTE IMERI FKUI lab, PBS 1X, Triton X-100 10%, Hematoxylin, and lithium carbonate.

qRT-PCR: Quick-RNA^TM^ Miniprep Plus Kit, Toyobo ReverTraAce qPCR RT Master Mix with gDNA remover, and SensiFAST^TM^ SYBR Lo-ROX One-Step Kit.

SEM: Silica gel.

IF: HNF4-alpha antibody [EPR3648] (ab92378 Abcam, UK), Recombinant anti-CEBP Alpha/CEBPA antibody [EP708Y] (ab40761 Abcam, UK), Goat F(ab’) 2 anti-rabbit IgG Fc (FITC) (ab6018 Abcam, UK).

### Methods

#### iPSC culture

*^16,^ ^17^*

iPSC cell line was thawed and cultured with the E8 culture medium in vitronectin-coated 12-well plates. Medium change was done every 2 days by removing 50% medium and adding equal volume of fresh medium. Microscopic observation and documentation was performed daily to evaluate colony morphology and confluency. Passage was done when confluency reaches 25–50% in each well. EDTA/Versene solution was used to disintegrate iPSC colony and to separate the colony from vitronectin after 4–5 minutes incubation in 37°C, 5% CO_2_ incubator. Cell scraper was used to ensure complete removal of iPSC from the vitronectin-coated well. iPSC suspension was collected in 5 ml complete medium with 10 µM ROCK inhibitor to halt the dissociative reaction. Split ratio was between 1:2 and 1:4.

### Making native liver scaffold

Native liver scaffold from New Zealand White Rabbit liver was made with decellularization methods by using multiple syringe injection. This method based on previous study that conducted.^14^ Ten lobules of liver were cut to a size of 1.5 cm x 1.5 cm with a thickness of 0.7–1 cm. The liver cubes were immersed in 0.001 M EGTA for 30 min and placed in a petri dish. A 1- ml syringe was fixed with a fixation device, with red wire attached with a toothpick on top of a styrofoam. The injection using fixated syringe was started with aquadest, continued with graded concentrations of 0.1%, 0.25%, 0.5%, 0.75%, and 1% SDS, and distilled water 25 times at the same point. Injections were carried out at the same site until the liver cube became clear. Further injection was repetitively done on 4–7 sites in the cube. The scaffolds were stored in 0.9% NaCl solution and placed in a freezer at −20°C (Supplementary Figure 1).

Prior to use in the study, the liver biological scaffolds were removed from the freezer and thawed in BSC. Then the scaffold was cutted into three parts with sterile surgical scissors and placed in a 12-well plate. UV sterilization was done in BSC for 1 hour prior to recellularization.

### Hepatocyte differentiation by using native liver scaffold

The recellularization stage was carried out by injecting 125,000 from total harvested iPSC with 1 ml syringe into 9 pieces of the liver biological scaffolds (Supplementary Figure 2). Since the number of total harvested MSCs were higher, each liver scaffold was recellularized with approximately 50,000 MSCs. After recellularization, the liver scaffold was cultured in a static 12-well plate culture with the medium change based on modified Blackford protocol^11^ for 21 days.

During the differentiation process, microscopic observations were made on the scaffold with an inverted microscope. After the differentiation process was carried out, the scaffold samples were retrieved on day 7 and day 21 for further analysis.

### Histology analysis for cell adhesion to ECM in scaffold and measurement of collagen area fraction

Hematoxylin eosin (HE) staining was carried out by means of preparations deparaffinized with xylol, rehydration by in decreasing graded alcohol (100%, 96%, 80%, and 70%). The preparations were then incubated in hematoxylin solution, then washed with running water for a short time, and continued with eosin incubation. The preparation was covered with entellan and cover slip.

MT staining was carried out by deparaffinizing preparations with xylol and rehydration by soaking in decreasing graded alcohol (100%, 96%, 80%, and 70%). The preparation was then put into Bouin’s fixative solution and soaked in Weigert hematoxylin solution and Biebrich scarlet acid fuchsin. The preparation is then immersed in phosphomolybdic acid and incubated in aniline blue. The results from the microphoto with Optilab were then analyzed with ImageJ software for quantification of the collagen area on MT staining.

### Cellular spatial distribution observed with scanning electron microscopy

The steps in this study were iPSC culture samples with a comparison of MSCs that were differentiated in liver biological scaffolds; histological technique was used to convert them into paraffin blocks. Then the sample was put into a container containing silica gel. Furthermore, the sample was coated with a gold layer and examined with the SEM model FEI Inspect F50 carried out at CMPFA FTUI. Data were collected by taking SEM photos at 5000X magnification.

### Immunohistochemical examination for hepatocyte marker albumin and CYP3A7

The preparation was immersed in 6% hydrogen peroxidase solution and incubated in protein block solution for 1 hour and washed with 0.1% PBST. Each preparation was then separately incubated with anti-albumin antibody (1:5000 dilution) and antiCYP3A4 (1:1000 dilution). Incubation was carried out at room temperature for 2 hours in a moist chamber. The preparations were then incubated with HRP-conjugated secondary antibody for 1 hour. The preparations were then added with 3-3’-diaminobenzidine (DAB) for 30 seconds and washed. The preparation was then counterstained with hematoxylin and dripped with lithium carbonate solution. The preparations were then observed with a light microscope and photographed with Optilab at 40x and 400x magnification, and then the data in the form of photographs were compared to the histological picture and the staining results between the treatment and control groups. The results of the photographs are then analyzed with ImageJ software for quantification of the area fraction in the IHC.

### Molecular analysis of hepatocyte differentiation from iPSCs or MSCs on rabbit liver biological scaffolds: albumin and CYP450 gene expression

The steps of RNA isolation from samples were carried out using the Quick-RNATM Miniprep Plus Kit (Zymo Research, USA). The RNA concentration was calculated using a NanoDrop spectrophotometer.

Complementary DNA (cDNA) synthesis using the protocol from Toyobo ReverTraAce qPCR RT Master Mix. The qRT-PCR in this study used the Applied Biosystems 7500 Fast machine and the SensiFASTTM SYBR Lo-ROX One-Step Kit reagent. The primer used was designed using NCBI and IDT PrimerQuest, with housekeeping gene using 18SS primer. The following is a list of the primer nucleotide sequences used in this study (Table 1).

**Table 1.** Primers nucleotide sequence

No	Gene	Accession code	Primer sequence
1	Albumin	NM_000477.7	F: TGCTTTGCCGAGGAGGGTAA R: AAGGCAGCTTGACTTGCAGC
2	CYP450	NM_017460.6	F: TCTTCCGGGGATATGGTGTGA R: CTCCACACTCCGCTTTCCCA
3	CK19		F: TCGACAACGCCCGTCTG R: CCACGCTCATGCGCAG

Each sample was repeated three times. The samples were then put into a PCR machine and run according to polymerase activation at 95°C, denaturation at 95°C, annealing at 60°C, and extension at 72°C. The CT values obtained were then processed using the Livak formula to determine the relative expression of albumin and CYP450 to the housekeeping gene 18SS gene.

### Determination of hepatocyte maturation from differentiated iPSCs or MSCs by qRT-PCR Cytokeratin-19 (CK-19) and immunofluorescence of hepatocyte transcription factors HNF4a and CEBPA

CK19 is an intermediate filament with a molecular weight of around 40 kDa. CK19 was detected in the primitive hepatic progenitor cells at the 4–10 weeks’ gestation. The expression of CK19 is diminished in mature hepatocytes.^12^ Primer nucleotide sequences of CK-19 are as follows: forward primer: TCGACAACGCCCGTCTG and reverse primer: CCACGCTCATGCGCAG.

The steps of the IF technique on samples of iPSC differentiation in liver biologic scaffolds were the preparations deparaffinized with xylol, rehydrated, and then incubated with namely anti-HNF4-α primary antibody (1:100 dilution) and anti-CEBPA (1:250 dilution). Incubation was carried out at 4°C for 2 hours in a moist chamber. The preparations were then washed with 0.1% PBST, followed by incubation of 1:2500 diluted anti-rabbit IgG Fc secondary antibody for 1 hour and washed with 0.1% PBST. The results of the IF staining were examined with a fluorescence microscope at HGRC IMERI FKUI.

### Data analysis

Data analysis in this study was carried out by statistical tests using Graph pad prism 9 software. Statistical analysis was performed on gene expression variables and histological quantitative analysis using ImageJ software (collagen area, albumin expression, CYP3A4). The normality test was carried out using the Shapiro test, and the homogeneity test was carried out using the Levene test. The p value <.05 indicates a significant difference between the two groups.

## Results

### Cell–ECM adhesion to scaffold

**Figure 1.**
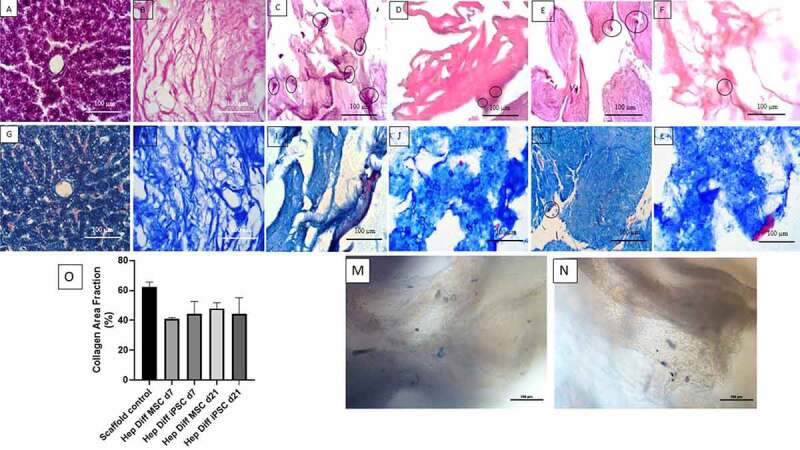
Histological features of hepatocyte differentiation in decellularized liver scaffold (Mag. 400x). A: HE-stained Liver control, B: HE-stained decellularized liver scaffold, C: HE-stained hepatocyte-differentiated MSC d7 in decellularized liver scaffold, D: HE-stained hepatocyte- differentiated iPSC d7 in decellularized liver scaffold, E: HE-stained hepatocyte-differentiated MSC d21 in decellularized liver scaffold, F: HE-stained hepatocyte-differentiated iPSC d21 in decellularized liver scaffold, G: Masson trichrome-stained liver control, H: Masson trichrome-stained decellularized liver scaffold, I: Masson trichrome-stained hepatocyte-differentiated MSC d7 in decellularized liver scaffold, J: Masson trichrome-stained hepatocyte-differentiated iPSC d7 in decellularized liver scaffold, K: Masson trichrome-stained hepatocyte-differentiated MSC d21 in decellularized liver scaffold, L: Masson trichrome-stained hepatocyte-differentiated iPSC d21 in decellularized liver scaffold cells suspected of being MSCs were clearly observed (marked with circles), M: Brightfield image from inverted microscope of hepatocyte-differentiated iPSC d7 in decellularized liver scaffold, N: Brightfield image from inverted microscope of hepatocyte-differentiated iPSC d7 in decellularized liver scaffold. Circles marked cell adherence to scaffold. O. Quantification collagen area using Image J measurement from hepatocyte differentiated MSCs (n = 3) or iPSCs (n = 3) in decellularized liver scaffold (%).

From Figure 1a, histology of normal liver showed tightly packed hepatocytes with eosinophilic cytoplasm and radial arrangement of hepatocytes with basophilic nuclei. In decellularized rabbit liver scaffolds with MSC differentiation d7 and d21, there was extracellular matrix with pores and hepatocyte-differentiated MSC cells attached to the scaffold with basophilic nuclei (see Figure 1b).

There was higher number of cellular adhesion to the scaffold with wider distribution in hepatocyte differentiated from MSCs than iPSCs (see Figure 1c,e vs Figure 1d,f). Homogenous cell morphology was frequently found in hepatocyte differentiated from MSCs d7 and d21 as showed in HE result (see Figure 1c,e). Diverse cell morphology ranging from flattened spindle shape to ovoid shape with heterogenous size was observed microscopically in hepatocyte derived from iPSCs on d7 and d21 (see Figure 1m,n). On d21, the attached cells seen were larger in size with distinction between the nucleus and cytoplasm appearing clearly both in hepatocyte-differentiated MSCs or iPSCs in decellularized liver scaffold (see Figure 1e vs figure 1f). From the results of the examination with HE staining, cell adhesion to ECM in scaffold was observed both in d7 and d21 hepatocyte-differentiated MSCs or iPSCs in decellularized liver scaffold with different profile.

Based on observations made on MT staining from Figure 1g, it was found that in normal liver parenchyma tightly packed with eosinophilic hepatocyte cytoplasm and blue-colored connective tissue. In decellularized liver scaffolds (Figure 1h), the extracellular matrix was composed of blue-colored collagen fibers with interspersed pores (appeared as hollow white spaces between fibers). In Figure 1i,k, the hepatocyte-differentiated MSC d7 and d21 with eosinophilic cytoplasm and blue-black-colored nuclei. They were seen on the surface of collagen fibers or filled the scaffold pores. In Figure 1j,k, the hepatocyte-differentiated iPSC d7 and d21 also were seen on the surface of collagen fibers or filled the scaffold pores.

In the measurements with ImageJ in Figure 1, it was found that the mean percentage of collagen area in the decellularized liver scaffold control was 62.56% with standard error of mean 1.12%. The mean percentage of collagen area in the hepatocyte-differentiated MSC d7 was 41.04% ± 0.42% and 47.88% ± 2.26% in hepatocyte-differentiated MSC d21. The mean percentage of collagen area in the hepatocyte-differentiated iPSC d7 was 44.43% ± 3.66% and 44.42% ± 4.77% in hepatocyte-differentiated iPSC d21. There was no statistical difference of collagen area result between groups (p > .5).

### Spatial cell distribution profile comparison between hepatocyte differentiated from iPSCs with MSCs in scaffold

**Figure 2.**
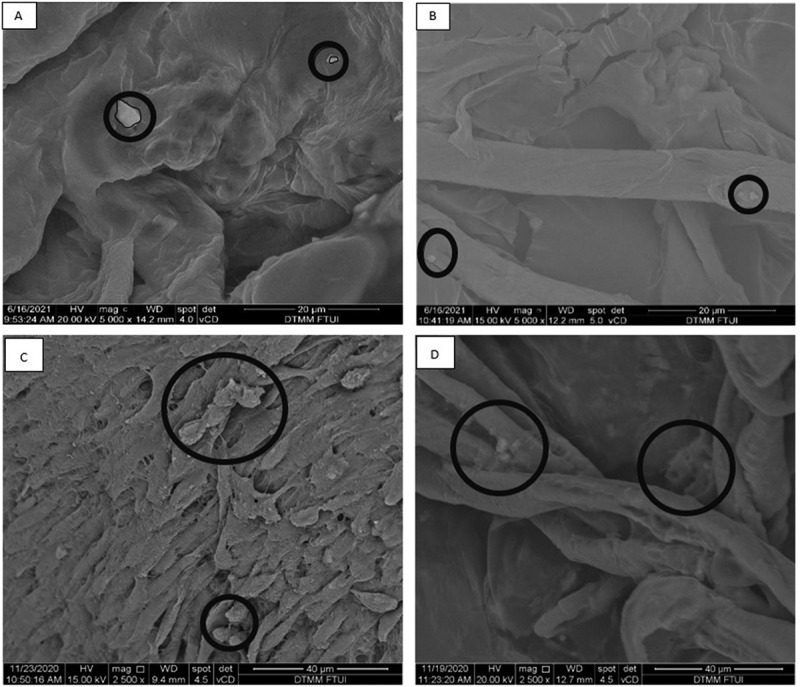
Representative SEM images of hepatocyte differentiation in decellularized liver scaffold (Mag. 5000x). A: hepatocyte-differentiated iPSC d7 in decellularized liver scaffold (n = 3), B: hepatocyte-differentiated iPSC 21 in decellularized liver scaffold (n = 3), C: hepatocyte-differentiated MSC d7 in decellularized liver scaffold (n = 3), F: hepatocyte-differentiated MSC d21 in decellularized liver scaffold (n = 3). Circles marked cell adherence to scaffold.

As a comparison in this study, the SEM images of hepatocyte differentiated from iPSCs and MSCs are showed in Figure 2a-d. The result showed a three-dimensional topographic image of the extracellular matrix collagen fibers with the presence of a few single-formation hepatocyte-differentiated iPSCs d7 attached to collagen fibers in decellularized liver scaffold (see Figure 2a). The distance between each single cell was far apart. The results were also seen on hepatocyte-differentiated iPSCs d21 in decellularized liver scaffold (see Figure 2b). In comparison, the results from Figure 2c-d showed that hepatocyte-differentiated MSC d7 and d21 distributed in different pattern than iPSC. The hepatocyte-differentiated MSCs d7 attached to the collagen fibers of the decellularized liver scaffold forming clusters of adherent cells in contact or in close proximity with each other (see Figure 2c) and more abundant. Similar results were also seen in hepatocyte-differentiated MSC d21. Different cellular spatial distribution in the decellularized liver scaffold between iPSCs and MSCs hepatic differentiation was described as single-isolated cell for hepatocyte-differentiated iPSCs and clustered-adjacent cells for hepatocyte-differentiated MSCs.

### Detection of albumin from hepatocyte-differentiated MSCs and iPSCs in decellularized liver scaffold

**Figure 3.**
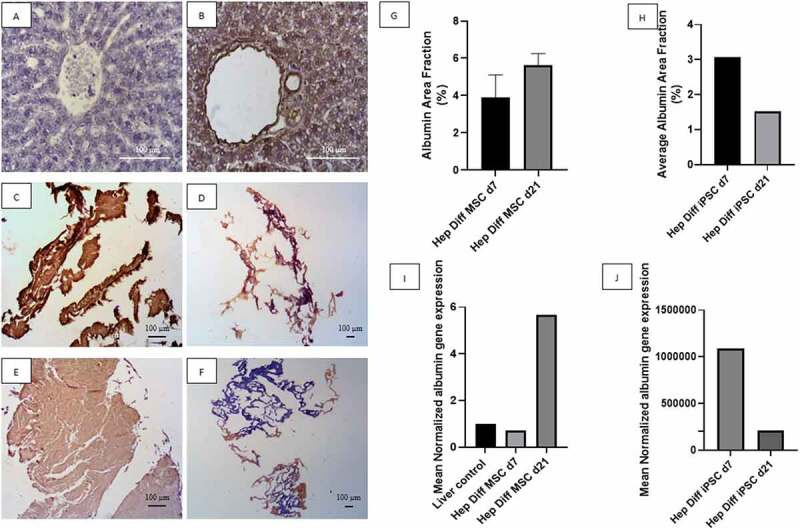
Albumin detection from hepatocyte differentiation in decellularized liver scaffold. A: IHC albumin liver negative control (Mag. 400x), B: IHC albumin liver positive control (Mag. 400x), C: IHC albumin hepatocyte-differentiated MSC d7 in decellularized liver scaffold (Mag. 40x), D: IHC albumin hepatocyte-differentiated iPSC d7 in decellularized liver scaffold (Mag. 40x), E: IHC albumin hepatocyte-differentiated MSC d21 in decellularized liver scaffold (Mag. 40x), F: IHC albumin hepatocyte-differentiated iPSC d21 in decellularized liver scaffold (Mag. 40x), G: Percentage of albumin area fraction using Image J measurement from hepatocyte-differentiated MSCs in decellularized liver scaffold (n = 3), H: Percentage of albumin area fraction using Image J measurement from hepatocyte-differentiated iPSCs in decellularized liver scaffold (n = 3), I: qRT-PCR mean normalized albumin gene expression from hepatocyte-differentiated MSCs in decellularized liver scaffold (n = 3), J: qRT-PCR mean normalized albumin gene expression from hepatocyte-differentiated iPSCs in decellularized liver scaffold (n = 3). Comparison between groups is not significant (p > .5).

Based on observations made on IHC staining with albumin antibody from Figure 3, positive immunoreactivity to albumin appeared as brown color in hepatocyte cytoplasm and less frequently in the nucleus or outside hepatocyte as shown in positive liver control (see Figure 3b). Negative immunoreactivity to albumin is shown in Figure 3a.

Low power field magnification captures the whole view of positive and negative area of albumin immunoreactivity against hepatocyte-differentiated MSCs and iPSCs in decellularized liver scaffold. Hepatocyte-differentiated MSCs in decellularized liver scaffold d7 (Figure 3c) revealed albumin-positive area depicted in brown almost covering the entire structure. Increment of albumin-positive area of hepatocyte-differentiated MSCs in decellularized liver scaffold d21 (Figure 3e) showed as bulkier brown stained structure. Hepatocyte-differentiated iPSCs in decellularized liver scaffold d7 (Figure 3d) showed partial albumin-positive area with more restricted patch of albumin-positive area in d21 (figure 3f). Quantification of albumin-positive area using image analysis is in line with the descriptive result. Mean albumin area fraction in hepatocyte-differentiated MSC in decellularized liver scaffold d7 was 3.88% ± 0.7% and 5.63% ± 0.3% in hepatocyte-differentiated MSC d21 (Figure 3g). The mean albumin area fraction is higher in hepatocyte-differentiated MSC in decellularized liver scaffold d21 than d7. There was no statistical difference of albumin area fraction result between d21 and d7 (p > .5). The mean of albumin area fraction in the hepatocyte-differentiated iPSC in decellularized liver scaffold d7 was 3.07% and 1.52% in hepatocyte-differentiated iPSC d21 (Figure 3h). There is a decrease in albumin area fraction d7 to d21 from hepatocyte-differentiated iPSC in decellularized liver scaffold.

qRT-PCR albumin result supports the IHC albumin result. Mean normalized albumin gene expression from hepatocyte-differentiated MSC in decellularized liver scaffold d7 was 0.73 and increased up to 5.68 in hepatocyte-differentiated MSC d21 (Figure 3i). This result confirms increment of albumin expression from hepatocyte-differentiated MSC in decellularized liver scaffold d7 to d21. Mean normalized albumin gene expression from hepatocyte-differentiated iPSC in decellularized liver scaffold d7 was 1085052.43 and decreased to 207919.66 in hepatocyte-differentiated iPSC d21 (Figure 3j).

### Identification of CYP450 from hepatocyte-differentiated MSCs and iPSCs in decellularized liver scaffold

**Figure 4.**
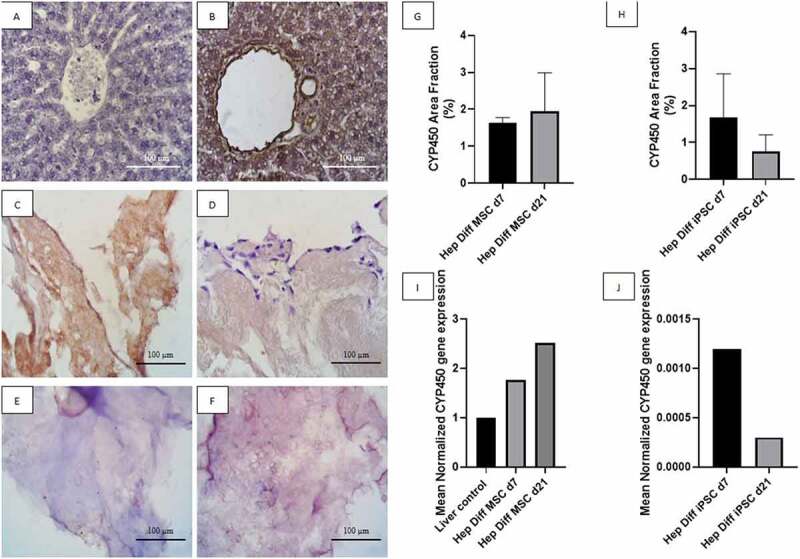
CYP450 identification from hepatocyte differentiation in decellularized liver scaffold (Mag. 400x). A: IHC CYP450 liver negative control, B: IHC CYP450 liver positive control, C: IHC CYP450 hepatocyte-differentiated MSC d7 in decellularized liver scaffold, D: IHC CYP450 hepatocyte-differentiated iPSC d7 in decellularized liver scaffold, E: IHC CYP450 hepatocyte-differentiated MSC d21 in decellularized liver scaffold, F: IHC CYP450 hepatocyte-differentiated iPSC d21 in decellularized liver scaffold, G: Percentage of CYP450 area fraction using Image J measurement from hepatocyte-differentiated MSCs in decellularized liver scaffold (n = 3), H: Percentage of CYP450 area fraction using Image J measurement from hepatocyte-differentiated iPSCs in decellularized liver scaffold (n = 3), I: qRT-PCR mean normalized CYP450 gene expression from hepatocyte-differentiated MSCs in decellularized liver scaffold (n = 3), J: qRT-PCR mean normalized CYP450 gene expression from hepatocyte-differentiated iPSCs in decellularized liver scaffold (n = 3). Comparison between groups is not significant (p > .5).

Based on observations made on IHC staining with CYP450 antibody from Figure 4, positive immunoreactivity to CYP450 appeared as brown color in hepatocyte cytoplasm as shown in positive liver control (see Figure 4b). Negative immunoreactivity to CYP450 is shown in Figure 4a as blue or purple stained nuclei with clear cytoplasm. High-power field magnification of positive and negative CYP450 immunoreactivity against hepatocyte-differentiated MSCs and iPSCs in decellularized liver scaffold showed larger details (see Figure 4c-f). Hepatocyte-differentiated MSCs in decellularized liver scaffold d7 (Figure 4c) revealed CYP450-positive cells in some area. Frequently found positive cells from hepatocyte-differentiated MSCs in decellularized liver scaffold d21 displayed as uniform light brown color covering almost the entire structure (Figure 4d) with the exclusion of some peripheral cells with blue-stained nuclei. Hepatocyte-differentiated iPSCs in decellularized liver scaffold d7 (Figure 4e) contained some CYP450-positive cells, which diminishes to a few CYP450-positive cells in d21 (Figure 4f). Quantification of CYP450-positive cells using image analysis is in line with the descriptive result. Mean CYP450 area fraction from hepatocyte-differentiated MSC in decellularized liver scaffold d7 was 1.63% ± 0.08% and 1.95% ± 0.6% in hepatocyte-differentiated MSC d21 (Figure 4g). There was no statistical difference of CYP450 area fraction result between d7 and d21 (p > .5). The mean of CYP450 area fraction in the hepatocyte-differentiated iPSC in decellularized liver scaffold d7 was 1.67% ± 0.53% and 0.75%± 0.2% in hepatocyte-differentiated iPSC d21 (Figure 4h). There is a decrease in CYP450 area fraction d7 to d21 from hepatocyte-differentiated iPSC in decellularized liver scaffold with no statistical significant difference.

qRT-PCR CYP450 result supports the previous result. Mean normalized CYP450 gene expression from hepatocyte-differentiated MSC in decellularized liver scaffold d7 was 1.76 and increased up to 2.52 in hepatocyte-differentiated MSC d21 (Figure 4i). This result confirms increment of CYP450 expression from hepatocyte-differentiated MSC in decellularized liver scaffold d7 to d21. Mean normalized CYP450 gene expression from hepatocyte-differentiated iPSC in decellularized liver scaffold d7 was 0.0012 and decreased to 0.0003 in hepatocyte-differentiated iPSC d21 (Figure 4j).

### Determination of hepatocyte maturation from differentiated iPSCs or MSCs in decellularized liver scaffold

**Figure 5.**
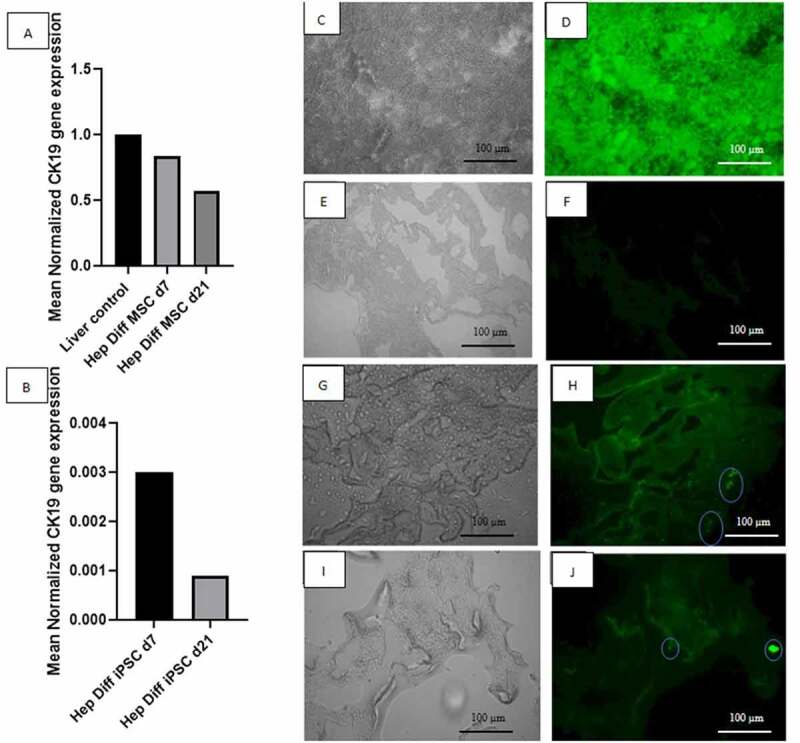
Determination of hepatocyte maturation stage by qRT-PCR CK-19 and IF HNF4-a and CEBPA. A: qRT-PCR mean normalized CK-19 gene expression from hepatocyte-differentiated MSCs in decellularized liver scaffold (n = 3), B: qRT-PCR mean normalized CK-19 gene expression from hepatocyte-differentiated iPSCs in decellularized liver scaffold (n = 3), C: Brightfield image from fluorescence microscope liver control, D: IF HNF4a liver-positive control, E: Brightfield image from fluorescence microscope decellularized liver scaffold, F: IF HNF4a decellularized liver scaffold control, G: Brightfield image from fluorescence microscope hepatocyte-differentiated iPSC d21 in decellularized liver scaffold, H: IF HNF4a hepatocyte-differentiated iPSC d21 in decellularized liver scaffold, I: Brightfield image from fluorescence microscope hepatocyte-differentiated iPSC d21 in decellularized liver scaffold, J: IF CEBPA hepatocyte-differentiated iPSC d21 in decellularized liver scaffold.

qRT-PCR CK-19 result is important to determine hepatocyte maturation stage from differentiated iPSCs or MSCs *in* decellularized liver scaffold. Mean normalized CK-19 gene expression from hepatocyte-differentiated MSC in decellularized liver scaffold d7 was 0.84 and decreased to 0.57 in hepatocyte-differentiated MSC d21 (Figure 5a). Mean normalized CK-19 gene expression from hepatocyte-differentiated iPSC in decellularized liver scaffold d7 was 0.0030 and decreased to 0.0009 in hepatocyte-differentiated iPSC d21 (Figure 5b). The rate of diminishing CK-19 expression during hepatocyte differentiation in decellularized liver scaffold is more prominent in iPSC than MSC (d7/d21 ratio = 3.33 vs. 1.47).

This result required further investigation with two hepatocyte transcription factors that regulate mature hepatocyte gene expression: HNF4a and CEBPA. HNF4a immunofluorescence result from hepatocyte-differentiated iPSC in decellularized liver scaffold d21 (Figure 5h) showed the presence of HNF4a-positive cells attached to the scaffold surface. CEBPA immunofluorescence result from hepatocyte-differentiated iPSC in decellularized liver scaffold d21 (Figure 5j) also showed the presence of CEBPA-positive cells attached to part of the scaffold.

## Discussion

Reconstruction of liver tissue engineering^18^ is a challenge for Indonesia, which categorized among lower middle income country in world bank list. Currently, one of the liver transplant centers available in Indonesia is Cipto Mangunkusumo National General Hospital as adjacent academic hospital of Faculty of Medicine, Universitas Indonesia, as the core institution where this study took place. The need of bioartificial liver from liver tissue engineering construct as bridge therapy for transplant waiting list patients who lived far from the liver transplant centers is pressing. This study is the beginning of developing bioartificial liver from liver tissue engineering studies at Faculty of Medicine, Universitas Indonesia, which is the frontier for stem cell and tissue engineering research in Indonesia. This is the first report of hepatocyte differentiation from iPSCs or MSCs in decellularized liver scaffold established by Department of Histology, Faculty of Medicine, Universitas Indonesia, in SCTE IMERI lab.

HE staining for iPSC differentiation showed fewer cells compared to MSC differentiation, with the scaffolds remaining intact in both cultures. In addition, cells attached to the liver biological scaffold were seen in iPSC differentiation but fewer than MSC differentiation. The initial number of cells seeded into the scaffold differ with lower number of iPSC than MSC. Initial seeding number of iPSCs to scaffold in this study is lower than in other studies. Those studies harvested iPSCs from culture after more than 80% confluence.^19–21^ In this study, prior to seeding, the iPSCs culture confluence nearly half from the reported value. Despite the theory of infinite proliferative capacity of iPSCs, harvesting lower than 80% confluence ultimately yields fewer iPSCs. This results in lower initial seeding number, which causes only a few iPSCs to attach to certain parts of the liver’s biological scaffold.

Collagen area that is occupied by hepatocyte-differentiated iPSCs or MSCs in this study represent cell–ECM interaction. Masson trichrome staining results indicated that the iPSCs differentiated in the scaffold interacts in different behavior than MSCs. The comparison shows that the hepatocyte-differentiated MSCs in scaffold d21 showed more reduction in collagen area than d7, while collagen area remains similar for hepatocyte-differentiated iPSC d21 and d7. Reduction in collagen area refers to the fact that more collagen area are occupied by growing number or size of cells. This could be due to proliferation and increase in cell size of the attached MSCs in the scaffold during in vitro hepatocyte differentiation. The similar collagen area percentage of hepatocyte-differentiated iPSC d21 and d7 indicates stable interaction between differentiating iPSCs and collagen fibers in the scaffold. This means the amount of collagen area whose spaces are occupied by the differentiating iPSCs remains constant during in vitro hepatocyte differentiation process. Collagen area difference could contribute to the different behavior of hepatocyte-differentiated iPSC compared with hepatocyte-differentiated MSCs. Heterogenous morphology and more restricted distribution of hepatocyte-differentiated iPSC in the scaffold in contrast to homogenous morphology and wider distribution of hepatocyte-differentiated MSC in the scaffold profile.

The results of SEM images further clarify the pattern of spatial cell distribution in the collagen fibers and scaffold pores. It shows that hepatocyte-differentiated MSC in the scaffold were cluster-adjacent cells and adhered more abundant to the collagen fibers than of hepatocyte-differentiated iPSC in the scaffold with single-cell-formation. In addition, the created scaffold retains a 3D structure that is useful as a microenvironment for the hepatocyte differentiation. The results of this study are unique compared with previous study SEM images, which showed more abundant iPSC adherence and denser iPSC filling the interconnected scaffold pores. Limitation of this study is the limited resource by which the recellularization technique was done manually by syringe injection of stem cell suspension to the decellularized liver scaffold. This causes recellularization to be abrupt and uneven as the pattern of single-cell-formation by hepatocyte-differentiated iPSC in the scaffold. Previous studies showed higher density of recellularization using perfusion pump with designated flow rate that facilitates balance spatial cell distribution in decellularized liver scaffold.^22–24^ This study shows hepatocyte-differentiated MSCs in the scaffold were cluster-adjacent cells and adhered more abundant to the collagen fibers. Plausible explanation may relate to the higher adhesion capacity from hepatocyte-differentiated MSC to collagen fibers in decellularized liver scaffold compared with hepatocyte-differentiated iPSCs. This result supports that of previous studies, which showed higher amount of hepatocytes differentiated from MSC with collagen fiber-based scaffold.^25–27^

The level of two hepatocytes markers albumin and CYP450 from hepatocyte-differentiated iPSC in decellularized liver scaffold is below that of hepatocyte-differentiated MSC. This was evidenced by a decreased trend in the area fraction analyzed with albumin and CYP3A4 IHC on hepatocyte-differentiated iPSC in decellularized liver scaffold d21. In contrast, hepatocyte-differentiated MSC in decellularized liver scaffold d21 showed an increase trend in the area fraction of albumin and CYP450. In addition, the results on albumin and CYP450 gene expression showed similar trends. These result are not in accordance with previous studies with comparable high level of albumin and CYP450 from hepatocyte-differentiated iPSC in decellularized liver scaffold.

Constant space occupying differentiating iPSCs with collagen fibers in the scaffold, abrupt, and uneven manual syringe injection recellularization are factors from this study which lead to low and decreased trends of albumin and CYP450 from hepatocyte-differentiated iPSC in decellularized liver scaffold. The distribution of hepatocyte-differentiated iPSC in decellularized liver scaffold that are far apart in this study have been identified by previous study to affect the communication between cells and the microenvironment, which causes the proliferation and differentiation processes to be less optimal. The multicellular mode of connection is a physical property of the interactions between cells. However, those physical properties have a significant impact on cell density, ligand receptor interactions, signal gradient processing, intracellular signal transduction, and the iPSC microenvironment.^28–30^ The next factor is the different degrees of differentiation between cells, where the differentiation process occurs faster in some parts of the scaffold, but in other parts of the scaffold, the differentiation process is slower. This may occur because many parts of the scaffold are not filled by cells or undergo cell death during the differentiation process, which affects differentiation and maturation into hepatocytes.

This study in in vitro hepatocyte differentiation protocol is one continuous set of iPSC differentiation in decellularized liver scaffold for 21 days with periodic hepatocyte induction medium change without perfusion pump machine. Previous studies showed higher hepatocyte differentiation efficiency by dividing each differentiation step, thus providing differences of culture conditions (enriched microenvironment), which set the stage for developmental milestones.^31,32^ Further selections of iPSC colony size, separation of undifferentiated iPSCs at the time of differentiation step, monitor iPSCs density before and after endoderm induction or further differentiation step.^33–36^ The microchip fluidic PMDS platform has been shown to recapitulate liver morphology and various liver functions for 4–6 weeks in vitro.^37,38^ In addition, previous study suggests arginylglycylaspartic acid (RGD) incorporated into scaffold designs to support cell attachment.^39^ Other approach is to involve these various techniques, including 3D co-culture with non-parenchymal cells (endothelial cells and mesenchymal cells) in the form of organoid.^40,41^

The positive result from this study is further maturation of hepatocyte stage from hepatocyte-differentiated iPSC in decellularized liver scaffold than hepatocyte-differentiated MSCs at transcriptional level. Diminishing CK-19 expression during hepatocyte differentiation in decellularized liver scaffold is more prominent in iPSC than MSC. If result confirms that there is positive signals for HNF4-α and CEBPA on hepatocyte-differentiated iPSC in decellularized liver scaffold d21.

Previous study elaborates the role of HNF4-a and CEBPA in hepatocyte differentiation. HNF4-α is a transcription factor that plays a central role in the differentiation of mature hepatocytes and forms the basis for building a network of transcription factors that regulate hepatic mRNA expression. The dependence on HNF4-α caused the transcriptional regulation of gene expression to stabilize as the liver transcription factor network increases in complexity during hepatocyte maturation. CEBPA is a member of the liver-specific CEBP transcription factor family and is correlated with hepatocyte maturation. CEBPA regulates the expression of albumin and AFP genes.^42–44^

CK-19, HNF4-A, and CEBPA expression from hepatocyte-differentiated iPSCs in decellularized liver scaffold appears to contradict the decreased trends of albumin and low CYP450 expression in this study.

Limitation of this study are fewer number of recellularized iPSCs than MSCs without prior optimization, lack of live/dead staining or proliferation marker Ki67 to evaluate cell survival in the scaffold, and absence of HNF4a and CEBPA immunofluorescence of the MSC-derived hepatocytes in the scaffold.

## Conclusion

Hepatocyte-differentiated iPSCs in decellularized liver scaffold differentiation is mature with lower cell–ECM adhesion, spatial cell distribution, albumin, and CYP450 than hepatocyte-differentiated MSC in decellularized liver scaffold.

## Supplementary Material

Supplemental Material
