# Supplementary figures and images for: Hepatocyte Differentiation from iPSCs or MSCs in Decellularized Liver Scaffold: Cell–ECM Adhesion, Spatial Distribution, and Hepatocyte Maturation Profile
Source: Organogenesis. 2022 Apr 17;18(1):2061263. doi: 10.1080/15476278.2022.2061263 (PMC9037523; doi:10.1080/15476278.2022.2061263)

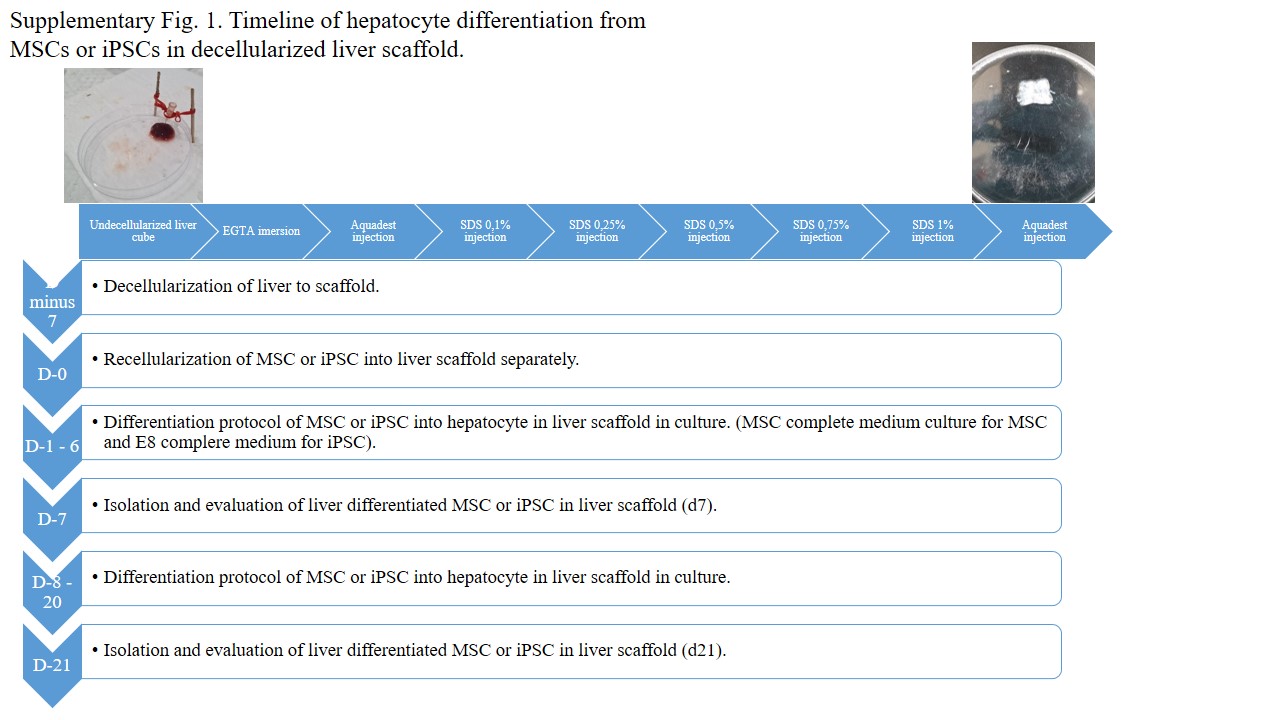

Supplement: Supplemental Material [file KOGG_A_2061263_SM0754.zip › Slide12.JPG]

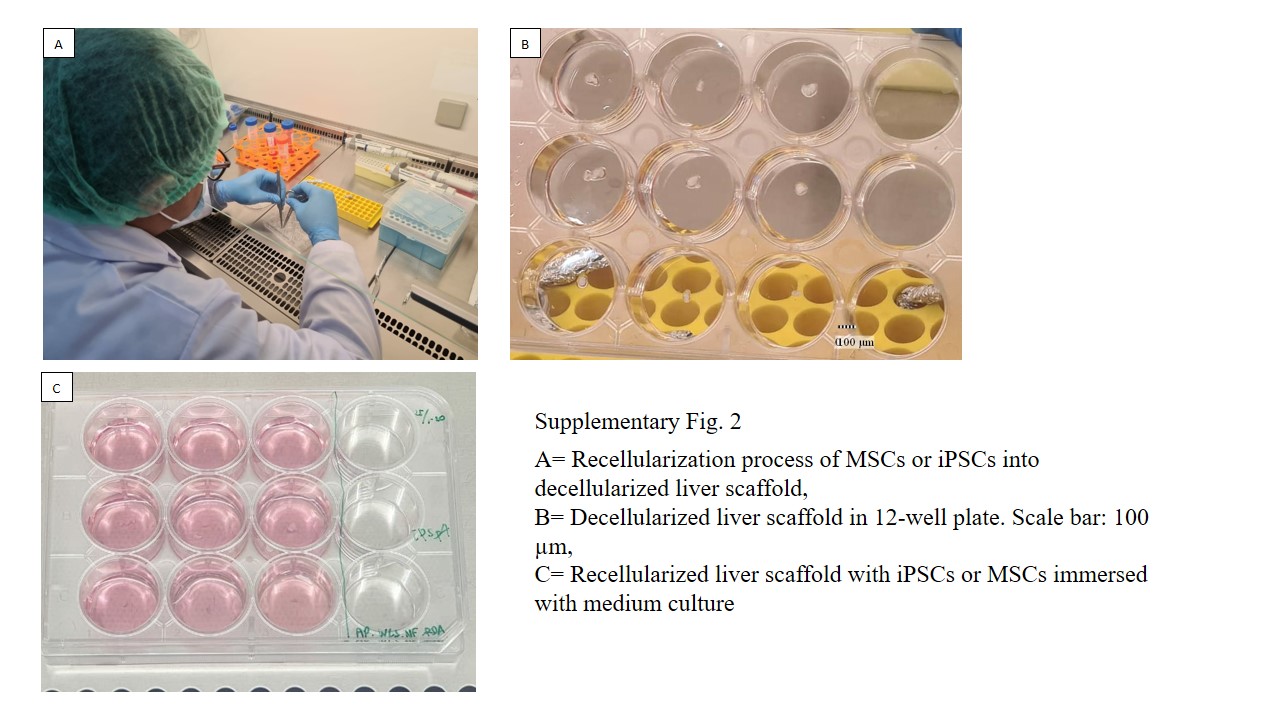

Supplement: Supplemental Material [file KOGG_A_2061263_SM0754.zip › Slide13.JPG]

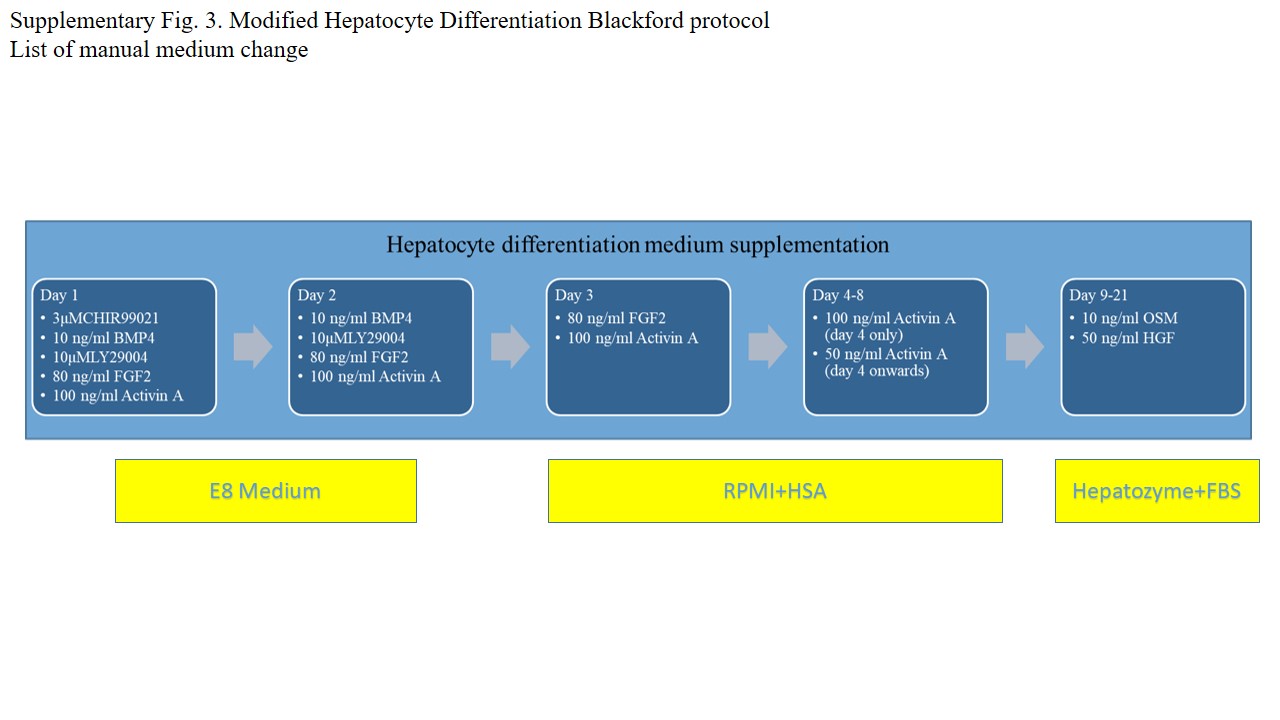

Supplement: Supplemental Material [file KOGG_A_2061263_SM0754.zip › Slide14.JPG]
